# Soft Tissue Mucosa-Associated Lymphoid Tissue Lymphoma: A Rare Case With an Interesting Presentation and Review of the Literature

**DOI:** 10.7759/cureus.48389

**Published:** 2023-11-06

**Authors:** Adnane Hammani, Othman Doghmi, El Mehdi Mahtat, Hicham El Maaroufi, Kamal Doghmi

**Affiliations:** 1 Hematology, Mohammed V Military Training Hospital, Faculty of Medicine and Pharmacy, Mohammed V University, Rabat, MAR; 2 Hematology, Ibn Sina University Hospital Center, Faculty of Medicine and Pharmacy, Mohammed V University, Rabat, MAR

**Keywords:** extranodal marginal zone malt lymphoma, 18f-fluorodeoxyglucose positron emission tomography (18f-fdg pet), immuno-chemotherapy, soft tissue lymphoma, mucosa-associated lymphoid tissue (malt) lymphoma

## Abstract

Soft tissue mucosa-associated lymphoid tissue (MALT) lymphoma is a rare type of marginal zone lymphoma. Herein, we report a case of a 61-year-old patient who developed soft-tissue marginal zone lymphoma in the right arm. He was treated with rituximab-chlorambucil with good metabolic response and no evidence of disease recurrence after one year of follow-up.

## Introduction

Extranodal marginal zone lymphoma (MZL), also known as mucosa-associated lymphoid tissue (MALT) lymphoma, is one of three subtypes of MZL and accounts for 7% of adult non-Hodgkin's lymphoma (NHL) and 70% of MZL [1]. Gastric MALT lymphoma is by far the most common clinical entity (70%), but other organs can be affected [2]. We report a case with soft tissue involvement of MZL.

## Case presentation

A 61-year-old patient was referred to the orthopedic department with a painful lump in the right forearm. Physical examination revealed a 4 cm red-colored mass on the posterior side of the right forearm and another mass in the right subclavicular region measuring 3 cm.

**Figure 1:**
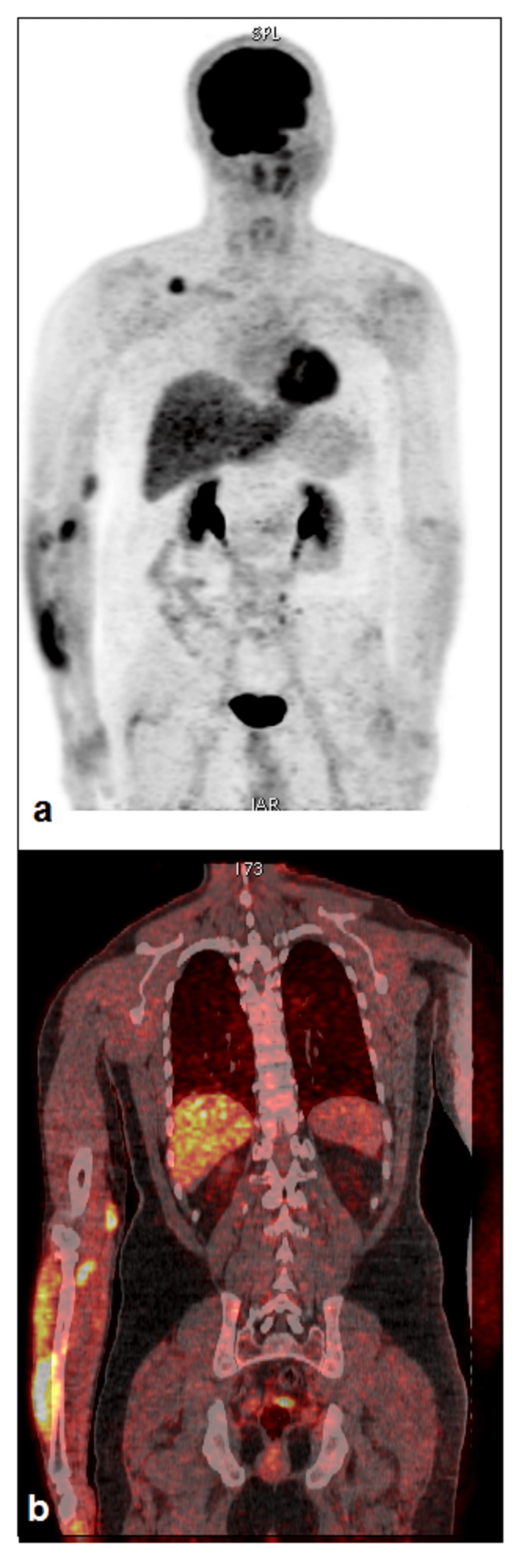
Maximum intensity projection (a) and coronal 18F-fluorodeoxyglucose (FDG) positron emission tomography/computed tomography fusion (b) showing FDG uptake throughout the soft tissues of the right upper limb and subclavicular region.

Laboratory test results showed the following: hemoglobin of 16.2 g/dl, white blood cells of 6.5 g/L, platelets of 200 g/L, lactate dehydrogenase oh 193 U/L (135-225), β2-microglobulin level of 2 mg/L (1-2.5), and C-reactive protein of 1 mg/L (<5). Serum protein electrophoresis was normal. Hepatitis B, C, HIV, and *Borrelia burgdorferi* serologies were negative, and screening for autoimmune disorders was also negative. Bone marrow biopsy was normal. Biopsy of the right arm mass showed infiltration of atypical cells expressing CD20 and a Ki-67 proliferation index of 15% and negative staining for CD5, CD23, CD10, CD3, and cyclin D, consistent with a marginal zone lymphoma phenotype. The patient has no B symptoms. Cervical, chest, and abdominopelvic computed tomography was normal. The patient had an 18F-fluorodeoxyglucose (FDG)-PET/CT that showed FDG-uptake foci throughout soft tissue of the distal part of the right arm (maximum standard uptake value (SUVmax) = 6.7), the proximal part of the right forearm (SUVmax = 6.9), the dorsal side of the right forearm (SUVmax = 10.2), and uptake in the right subclavicular region (SUVmax = 5), without another FDG uptake on the rest of the body (Figure 1). He was therefore staged IE.

He received induction treatment with daily chlorambucil of 6 mg/m2 orally for 42 consecutive days, then 6 mg/m2 per day for two weeks every four weeks (one cycle) for up to four cycles. The treatment was associated with rituximab 375 mg/m2 administered intravenously on days one, eight, 15, and 22 during the induction phase and on day one of each of the subsequent chlorambucil cycles.

**Figure 2:**
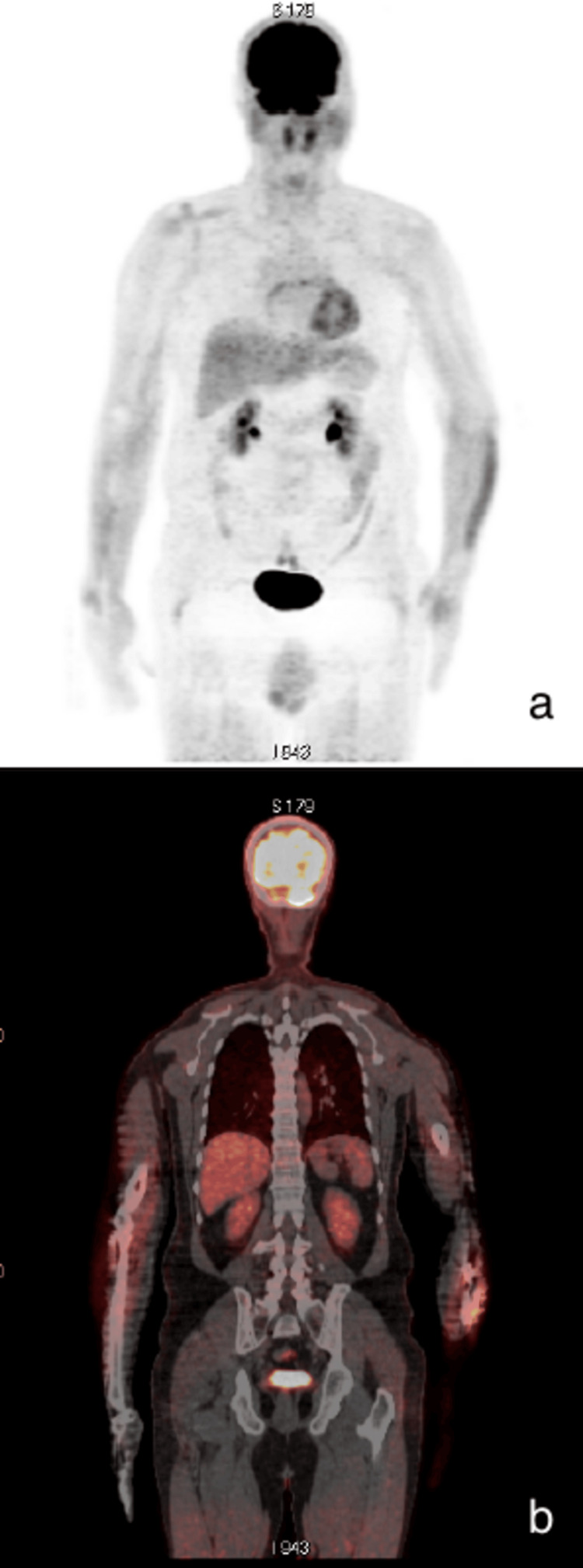
Maximum intensity projection (a) and coronal 18F-fluorodeoxyglucose positron emission tomography/computed tomography fusion (b) showing a total regression of the hypermetabolic lesions.

Physical examination during the two first months, six months, and one year after treatment revealed no palpable residual mass. One year after treatment, FDG PET/CT showed a total regression of the hypermetabolic lesions suggesting a complete response to therapy (Figure 2).

## Discussion

Extranodal MZL is one of three distinct clinical subtypes of MZL; it represents 7% of NHL. The most frequently affected organ is the stomach, followed by ocular adnexa, salivary glands, and lungs [3]. They are usually associated with chronic immune stimulation secondary to infection in most cases. Gastric MALT lymphoma is associated with *Helicobacter pylori* infection, cutaneous MALT with *Borrelia burgdorferi*, small intestinal MALT with *Campylobacter jejuni*, and ocular MALT with *Chlamydia psittaci* [4].

Soft tissue MALT lymphoma, as in our case, is extremely rare and to our knowledge, there is no consensus on the treatment of this entity in the literature.

Yonal-Hindilerden et al. reported a 57-year-old woman with a MALT lymphoma infiltrating the muscle in the left arm with bilateral axillary lymph nodes; treated with six cycles of standard-dose rituximab, cyclophosphamide, doxorubicin, oncovin and prednisone (R-CHOP) with two additional infusions of rituximab. She also received radiotherapy to her left arm at a dose of 30 Gy. Treatment assessment showed no evidence of residual disease [5]. This case suggests that MALT lymphoma of soft tissue can be treated with intensive chemotherapy used in some aggressive NHL. Edwards-Bennett et al. reported that a 67-year-old woman developed MZL of the right triceps one month after influenza vaccination at the same site. She was treated with involved field radiotherapy (IFRT) at a dose of 4000 cGy divided into 20 sessions with good response and no signs of disease after one year of follow-up [6]. In the phase III study by International Extranodal Lymphoma Study Group 19 (IELSG-19), 132 patients with MALT lymphoma were treated with rituximab and chlorambucil. The study led to an improvement in the quality of remission and significantly prolonged event-free survival (EFS) and progression-free survival (PFS) with less toxicity [7].

The available data on therapeutic protocols are very limited. In our case, the patient was treated with rituximab associated with chlorambucil, a protocol considered less intense with less toxicity, and we have obtained a complete response on assessment by FDG-PET/CT.

FDG-PET/CT imaging has achieved an unprecedented reputation in the field of lymphoma. The extent of MALT lymphoma on FDG-PET/CT poses two challenges: low FDG-avidity of extranodal lesions MALT lymphoma and their tendency to be located in tissues with physiologic FDG-uptake, making interpretation difficult [8].

## Conclusions

There is limited data regarding the prognosis and treatment of MALT Lymphoma of soft tissue. In our case, we conclude that rituximab and chlorambucil can be an effective treatment for MALT lymphoma of soft tissue and the 18F-FDG PET/CT can be used as an imaging tool for the response assessment.
